# Monotreme *glucagon-like peptide-1* in venom and gut: one gene – two very different functions

**DOI:** 10.1038/srep37744

**Published:** 2016-11-29

**Authors:** Enkhjargal Tsend-Ayush, Chuan He, Mark A. Myers, Sof Andrikopoulos, Nicole Wong, Patrick M. Sexton, Denise Wootten, Briony E. Forbes, Frank Grutzner

**Affiliations:** 1 Robinson Research Institute, School of Biological Sciences, The University of Adelaide, South Australia, 5000, Australia; 2 School of Applied and Biomedical Sciences, Federation University Australia, Mount Helen, Victoria, 3353, Australia; 3 Department of Medicine, Austin Health, The University of Melbourne, Heidelberg, Victoria 3084, Australia; 4 Monash Institute of Pharmaceutical Sciences and Department of Pharmacology, Monash University, Parkville, Victoria, 3052, Australia; 5 School of Medicine, Flinders University, Bedford Park, South Australia, 5042, Australia

## Abstract

The importance of Glucagon like peptide 1 (GLP-1) for metabolic control and insulin release sparked the evolution of genes mimicking GLP-1 action in venomous species (e.g. Exendin-4 in *Heloderma suspectum* (gila monster)). We discovered that platypus and echidna express a single GLP-1 peptide in both intestine and venom. Specific changes in GLP-1 of monotreme mammals result in resistance to DPP-4 cleavage which is also observed in the *GLP-1* like *Exendin-4* expressed in *Heloderma* venom. Remarkably we discovered that monotremes evolved an alternative mechanism to degrade GLP-1. We also show that monotreme GLP-1 stimulates insulin release in cultured rodent islets, but surprisingly shows low receptor affinity and bias toward Erk signaling. We propose that these changes in monotreme GLP-1 are the result of conflicting function of this peptide in metabolic control and venom. This evolutionary path is fundamentally different from the generally accepted idea that conflicting functions in a single gene favour duplication and diversification, as is the case for *Exendin-4* in gila monster. This provides novel insight into the remarkably different metabolic control mechanism and venom function in monotremes and an unique example of how different selective pressures act upon a single gene in the absence of gene duplication.

Discovery of the so called *incretin* effect, where ingested glucose leads to enhanced insulin release compared to intravenous glucose administration, revealed the existence of insulin release factors in the gut^1^. In humans gastric inhibitory polypeptide (GIP) and glucagon-like peptide 1 (GLP-1) are the only known insulin stimulating incretin hormones and are released from intestinal cells after food intake^2^,^3^,^4^,^5^. GLP-1 potentiates glucose-stimulated insulin secretion from pancreatic beta cells and promotes their survival. GLP-1 acts through the GLP-1 receptor (GLP-1R) expressed in the pancreas, brain and other peripheral tissues^6^. Both GIP and GLP-1 peptides are rapidly degraded in the gut and serum by the peptidase dipeptidyl peptidase-4 (DPP-4)^7^.

During vertebrate evolution the incretin system is highly conserved. Orthologs of the genes encoding the ligands (*GCG, GIP*), their receptors (*GLP-1R, GIPR*) and *DPP-4* have been identified in a range of vertebrate species (reviewed in refs 8, 9, 10). Although the *GCG* is expressed by all vertebrates the role of GLP-1 as an incretin was apparently acquired after the divergence of tetrapods from fish^11^. It is only recently that other functions for GLP-1, for example in the brain and cardiovascular system, have also been recognized^12^,^13^.

Components of the incretin and insulin systems also play a role in venomous species, which capitalize on the rapid and debilitating effects of low blood glucose when targeting prey. Examples of the evolution of venom components affecting glucose homeostasis include a *Glp-1* like gene *exendin-4* expressed in the lizard *Heloderma suspectum* (Gila monster) venom (now underpinning a type 2 diabetes drug^14^,^15^), a fish-like insulin mimetic in cone snail venom^16^ and the GLP-1 degrading enzyme DPP-4 in venom of various snake species^17^. Acquisition of novel gene function, such as the roles of GLP-1 and insulin variants in venom, is generally thought to occur through gene duplication followed by functional diversification. The evolutionary origin of *exendin* genes (*exendin* 1–4) has not been fully resolved but it seems likely that *exendins* evolved by duplication from a glucagon-like peptide gene precursor^14^.

We investigated the incretin hormone system in monotremes because of their phylogenetic position as the most basal lineage amongst extant mammals. In addition monotremes feature extraordinary changes in their digestive system, which has led to loss of genes involved in protein degradation and metabolic control^18^,^19^, suggesting unique mechanisms of metabolic homeostasis. Also, monotremes produce venom during breeding season and in platypus there is an elaborate venom delivery system, apparently used in competition for mating^20^,^21^. These observations prompted us to investigate the expression and function of incretin hormones in monotremes. To our surprise we discovered that only a single *GCG* encodes a GLP-1 peptide with two functions, one in venom and the other in the gut. Detailed *in vitro* characterisation of monotreme GLP-1 peptides revealed evolutionary signatures that can be explained by selective conflict as a result of the recruitment of this molecule into venom.

## Results

### Sequence variations in monotreme Gcg genes are present in regions known to be important for the regulation of protein function

**Figure 1:**
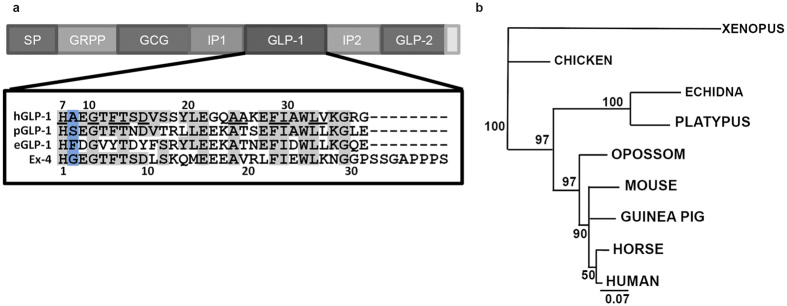
Identification and characterisation of monotreme *Gcg genes* and GLP-1 peptides. (**a**) Alignment of human, platypus, echidna and gila monster (Ex-4) GLP-1 sequences highlights hGLP-1 residues involved in hGLP-1R binding (underlined)^26^,^27^,^28^,^29^. The DPP-4 target site is highlighted in blue. Residues identical to those of hGLP-1are boxed (grey). (**b**) Phylogenetic reconstruction (Neighbor Joining, MEGA4) based on preproglucagon amino acid sequences of nine vertebrate species. The preproglucagon peptide domains: signal peptide (SP), glicentin-related polypeptide (GRPP), glucagon (GCG), intermediate peptides-1 and -2 (IP-1 and IP-2), glucagon like peptides 1 and -2 (GLP-1 and GLP-2).

*Gcg*, *Glp-1r*, *Gip* and *Dpp-4* genomic sequences were identified in the platypus genome database. At the beginning of this study there were no reported transcript sequences for these genes and some of the genomic sequences were incomplete. In this study for the first time cDNA sequences encoding platypus and echidna preproglucagon (encoded by the *GCG* gene) were identified (Supplementary Table S2). Phylogenetic analysis of the preproglucagon amino acid sequence in vertebrates revealed expected tree topology but also highlights sequence divergence in the monotreme lineage (Fig. 1b). The proglucagon peptide (Fig. 1a) includes a signal peptide (SP), glicentin-related polypeptide (GRPP), glucagon (GCG), intermediate peptides-1 and -2 (IP-1 and IP-2), glucagon like peptides 1 and 2 (GLP-1 and GLP-2), and is proteolytically processed into the mature glucagon-like peptides. While therian mammal *GCG* genes encode identical GLP-1 protein sequences, there are significant changes in the platypus *Gcg* ortholog. Importantly the inferred sequence of the platypus GLP-1 peptide (pGLP-1) differs in 11 of the 30 amino acids (37%) compared to human GLP-1 (hGLP-1, Fig. 1a, as reported previously^8^). A smaller difference was seen between platypus and human GLP-2 (30%) or glucagon peptides (20%) (Supplementary Fig. S1). Notably we also discovered specific changes in the DPP-4 cleavage site in pGLP-1 (pGLP-1, Ala^8^ to Ser) (Fig. 1a) and in the platypus GIP peptide (Supplementary Fig. S1, Ala^2^ to Ser), whereas there was no change to the cleavage sites in platypus glucagon or GLP-2 (Supplementary Fig. S1). To investigate if this change is also present in the echidna GLP-1 (eGLP-1) we cloned the echidna *Gcg* (*eGcg*) transcript and found a different amino acid at residue eight (Ala^8^ to Phe) in the eGLP-1 DPP-4 cleavage site (Fig. 1a), as well as a total of 17 differences from hGLP-1 of the 30 amino acids (57% changed). We also saw differences in echidna GLP-2 (pGLP-2) from hGLP-2 (45% changed), and in echidna glucagon from human glucagon (17% changed) (Supplementary Fig. S2). However, there was no change to the DPP-4 cleavage sites in both peptides. Remarkably, GLP-1 peptide sequence comparisons revealed a total of 12 differences between the two monotreme sequences (i.e. 40% of the sequence), indicating not only divergence from other mammals but major divergence within the monotremes, which separated only 17–48 Million years ago^22^ (Fig. 1b). These results raise fundamental questions about stability and potency of monotreme GLP-1.

### Monotreme Gcg and Dpp-4 exhibit similar tissue expression patterns to other mammals, but both genes are also expressed in venom

**Figure 2:**
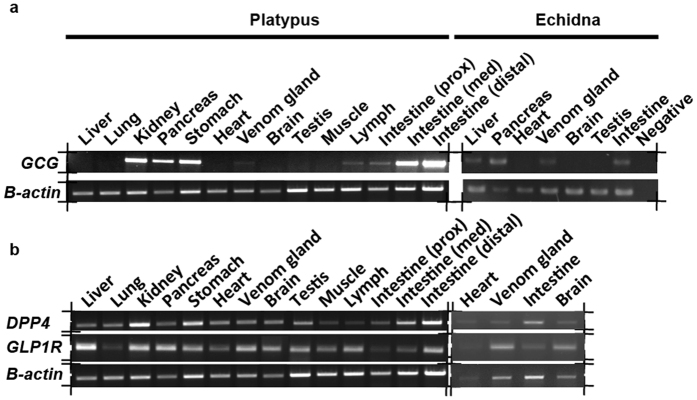
Expression of platypus and echidna *Gcg, Dpp-4* and *Glp-1r* in different tissues assessed by RT-PCR. (**a**) RT-PCR amplified *Gcg*, (**b**) *DPP-4* and *GLP-1R* showing expression in a range of tissues including venom gland. Beta actin was used as a positive control. These gel photos have been cropped as indicated.

Expression analysis showed that platypus and echidna *Gcg*, *Glp-1r* and *Dpp-4* tissue expression is similar to other mammals (Fig. 2)^23^,^24^, suggesting they play a similar role in monotremes. Surprisingly, both *Gcg* and *Dpp-4* genes are also expressed in platypus and echidna venom (Fig. 2). In monotremes it is the same *Gcg* gene encoding GLP-1 that is expressed in gut and venom. This is in contrast to *Heloderma suspectum*, where the *Gcg* is expressed in gut and *Exendin-4* in venom^15^.

### Monotreme GLP-1 peptides are resistant to cleavage by human DPP-4 and human serum components but are cleaved in platypus and echidna sera, independently of DPP-4 activity

**Figure 3:**
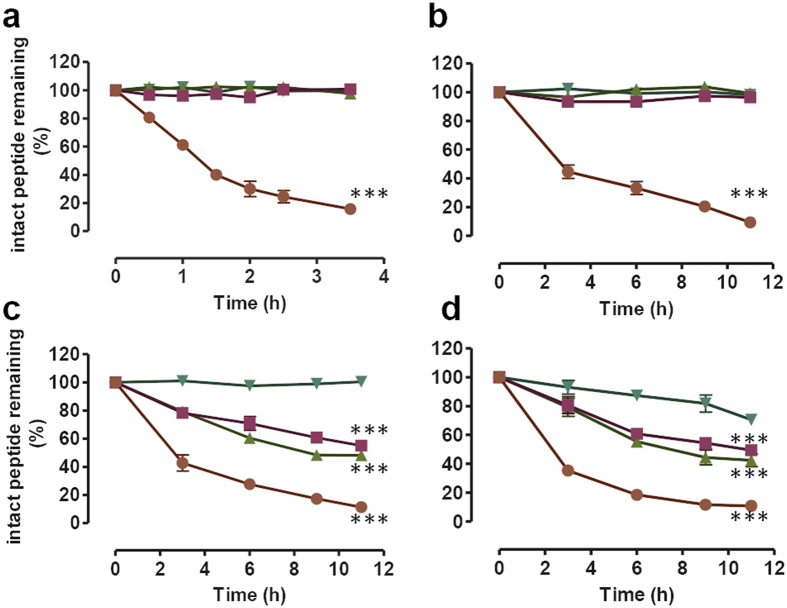
Degradation of hGLP-1 (orange circles), pGLP-1 (pink squares), eGLP-1 (green triangles) and Ex-4 (turquoise inverted triangles) at different incubation times by purified human DPP-4 enzyme (**a**), human serum (**b**), platypus serum (**c**) and echidna serum (**d**) determined by measuring the area under the curve of the intact peptide following rHPLC analysis. All values represent means ± S.E.M. (n = 3). ***Statistically significant, *P* < 0.001 peptide remaining at last time point compared with starting peptide concentration.

Sequence alignments between human, platypus and echidna GLP-1 proteins (Fig. 1) revealed substitutions at the known DPP-4 cleavage site (Ala^8^ in human) to Ser or Phe in platypus and echidna respectively, predicting that DPP-4 cleavage could be affected. To test if the specific amino acid changes at the DPP-4 cleavage site in pGLP-1 and eGLP-1 result in resistance to degradation, we compared their cleavage to that of human GLP-1 (hGLP-1) and the DPP-4 resistant exendin-4 (Ex-4) (Fig. 3). Incubation of the peptides with purified human DPP-4 resulted in rapid degradation of hGLP-1 (50% reduction of intact peptide within 1 hour) but not Ex-4. Significantly, both echidna and platypus GLP-1 were not degraded by human DPP-4 (Fig. 3a), confirming that monotreme GLP-1 is resistant to DPP-4 cleavage. Next we investigated stability in human serum. In albumin-depleted human serum, platypus and echidna GLP-1 and Ex-4 remained stable whereas hGLP-1 was rapidly degraded (Fig. 3b). To investigate if monotremes employ a different way to break down GLP-1, we measured cleavage in platypus and echidna serum. Surprisingly, degradation of platypus and echidna GLP-1 was observed when incubated in platypus and echidna sera (Fig. 3c,d). Degradation was slower than for hGLP-1 but clearly measurable, with less than 50% uncleaved pGLP-1 and eGLP-1 remaining after 11 hours of incubation (Table 1). Interestingly, Ex-4 was cleaved slowly in echidna serum but remained intact in platypus and human sera.

**Figure 4:**
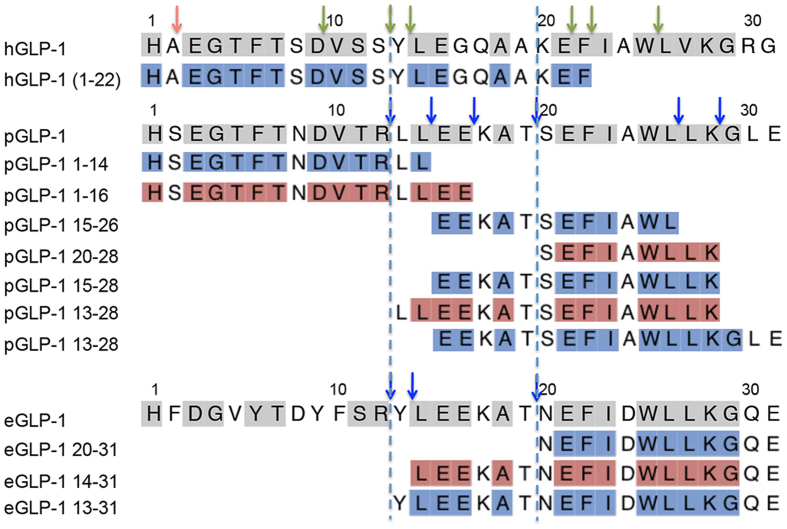
Amino acid sequences of cleavage products predicted by MALDI-Mass spectrometry. Degradation of hGLP-1, pGLP-1 and eGLP-1 in echidna serum for seven hours was monitored by RP-HPLC, fragments were collected and analysed by mass spectometry. Known cleavage sites of DPP-4 and NEP24.11 are shown above the hGLP-1 sequence by red and green arrows, respectively. Identified cleavage sites in pGLP-1 and eGLP-1 are highlighted by blue arrows.

DPP-4 is not the only enzyme that can degrade GLP-1. Human neural endopeptidase (NEP24.11), for example also cleaves GLP-1 but utilizes different target sites within the peptide^25^. To further investigate whether monotremes evolved a DPP-4 independent pathway to degrade GLP-1, we firstly confirmed the presence of DPP-4 in platypus and echidna sera using a synthetic DPP-4 peptide as a substrate and a DPP-4 inhibitor to prevent this cleavage (Supplementary Fig. S3), thus showing that cleavage was due to DPP-4. Despite the presence of DPP-4 inhibitor we were able to detect cleavage of both monotreme GLP-1 peptides (Supplementary Fig. S4), showing that a different enzyme(s) must be responsible for their cleavage in monotreme sera. To gain further insight into the mechanism of degradation we used mass spectrometry to analyse the pGLP-1 and eGLP-1 cleavage products. We saw cleavage products that suggested trypsin or chymotrypsin-like activity with cleavage after basic and hydrophobic residues (Fig. 4). Together this supports the idea that in monotremes a DPP-4 independent system has evolved to regulate GLP-1 half-life and activity.

### Monotreme GLP-1 peptides bind with lower affinity to the GLP-1 receptor than human GLP-1

**Figure 5:**
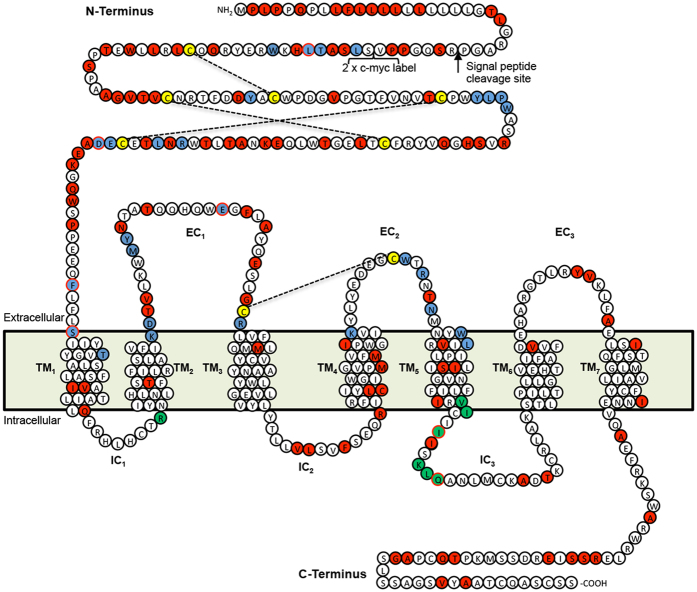
Schematic diagram of the pGLP-1R highlighting residues equivalent to hGLP-1R important for structure and function. Cysteine residues involved in disulphide bonds, denoted by dashed lines, are highlighted in yellow. Residues vital for ligand recognition and binding are highlighted in blue^12^,^29^,^52^. Residues important for receptor signalling through interaction with Gs-proteins are highlighted in green boxes^29^. Residues different between hGLP-1R and pGLP-1R are highlighted in red. Where a residue is marked blue or green but is different to hGLP-1 it is highlighted by a red circle. The putative signal peptide cleavage site is depicted with a black arrow.

**Figure 6:**
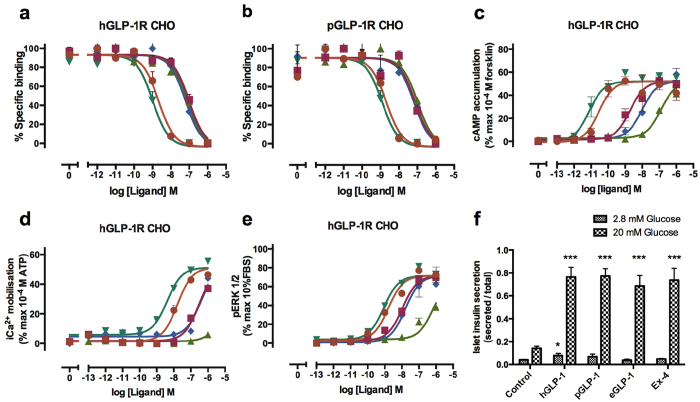
hGLP-1R and pGLP-1R binding and hGLP-1R signalling. Characterisation of the binding of hGLP-1 (orange circles), pGLP-1 (pink squares), eGLP-1 (green triangles), Ex-4 (green turquoise inverted triangles) and OXM (blue diamonds) in competition with radiolabeled ^125^I-hGLP-1 (7–36) in FlpInCHO cells stably expressing hGLP-1R (**a**) or pGLP-1R (**b**). Data are normalized to the maximum ^125^I-hGLP-1 (7-36) of each data set. cAMP accumulation (**c**), Ca^2+^ mobilization (**d**) and ERK1/2 phosphorylation (**e**) was measured using FlpInCHO cells stably expressing hGLP-1R. Data were normalized to the maximal response induced by 100 μM forsklin (cAMP), 100 μM ATP (Ca^2+^) or 10% FBS (ERK1/2) respectively. Data were analysed with a three-parameter logistic equation as described previously^47^. (**f** ) Glucose-induced insulin release stimulated with or without 100 nM each peptide in the presence of either 2.8 mM or 20 mM of glucose. (**g**) Webs of bias generated to quantify and compare signalling bias as described in the methods section. Data are presented on a log scale. All values are the means ± S.E.M from at least three independent experiments performed in triplicate. *Statistically significant at *P* < 0.05 versus negative control group without peptide, ***Statistically significant at *P* < 0.001 versus negative control group without peptide.

We then asked how changes in pGLP-1 and eGLP-1 affect binding and activation of the GLP-1 receptor (GLP-1R). All of the known key hGLP-1 residues (underlined in Fig. 1a) involved in binding to the human GLP-1R (hGLP-1R) core and four of the six C-terminal residues (excepting Ala^25^ to Thr and Val^33^ to Leu) involved in binding to the hGLP-1R N-terminal domain are conserved in pGLP-1^26^,^27^,^28^. Interestingly, in echidna GLP-1 there is less conservation of the receptor binding residues with additional changes at the N-terminal receptor binding Phe^12^ (conservatively substituted to Tyr) and Asp^15^ (changed to Glu) residues. hGLP-1 Gly^22^, involved in kinking of the helix, is a Glu in the extended Ex-4 helix, leading to different modes of interaction with the GLP-1R^29^. The same substitution is seen in the monotreme GLP-1 sequences (Fig. 1a). The platypus GLP-1R amino acid sequence (deduced in this study from a sequenced cDNA transcript) is similar to the hGLP-1R (76% identity compared to hGLP-1R), including conservation of the residues important for ligand binding (Fig. 5). The pattern of pGLP-1R expression (Fig. 2b) is also similar to other mammals^30^. Receptor binding assays on hGLP-1R overexpressing cells showed that, compared to hGLP-1 and Ex-4 both platypus and echidna GLP-1 peptides have lower affinity for the human receptor (Fig. 6a, Table 2). hGLP-1 has an almost identical affinity for the platypus GLP-1R (pGLP-1R) and the human receptor, but unexpectedly both monotreme GLP-1 peptides had a significantly lower affinity than hGLP-1 for the pGLP-1R (Fig. 6b, Table 2). For both receptors monotreme GLP-1 peptides were equipotent with the GLP-1R agonist oxyntomodulin (OXM).

### Monotreme GLP-1 peptides are less potent in their activation of the GLP-1 receptor

We then investigated if this difference in affinities translates into a difference in activation of the GLP-1 receptor. As expected monotreme GLP-1 peptides showed significantly less potency than hGLP-1 in assays measuring cAMP accumulation, Ca^2+^ mobilization and ERK1/2 phosphorylation acting through both human and platypus receptors (Fig. 6c–e). eGLP-1 showed a markedly lower potency at both human and platypus receptors that was even lower than OXM. Differences in the structure of monotreme GLP-1 peptides compared with hGLP-1 could account for the lower affinity for the receptor. Circular dichroism spectroscopy (CD) on Ex-4 and hGLP-1 yielded results similar to previously published data^26^ and showed that all peptides utilized were folded correctly (Supplementary Fig. S5). All peptides retained significant helical content, although pGLP-1 had more and eGLP-1 had slightly less than hGLP-1. As has been seen with Ex-4^26^, a difference in helical content can result in a different mode of interaction with GLP-1R.

### Monotreme GLP-1 elicits a different signalling cascade compared to hGLP-1 when binding to the GLP-1 receptor

Closer examination of potencies in receptor activation revealed differential signalling bias for monotreme GLP-1 peptides in comparison to that elicited by hGLP-1. Distinct signalling bias arising through activation of the GLP-1R by different ligands (including OXM) has recently been established and may, at least in part, underlie differences in the physiological profile of naturally occurring ligands of the GLP-1R^12^. Indicators used to determine the signalling profile of peptides include cAMP and intracellular Ca^2+^ mobilisation, which are involved in promotion of insulin release, and pERK1/2 that is part of the mitogenic signalling pathways activated via the GLP-1R^12^. Intriguingly, both the platypus and echidna GLP-1 peptides displayed a distinct pattern of signalling in comparison to hGLP-1 and the clinically approved mimetic Ex-4, which was apparent at both the human and platypus GLP-1 receptors (Fig. 6, Supplementary Figs S6 and S7, Table 3). The signalling profile of the monotreme GLP-1 peptides closely matched that of OXM with a bias towards pERK1/2, and to a lesser extent iCa^2+^, relative to cAMP (Fig. 6, Supplementary Figs S6 and S7, Table 3), although the bias towards calcium mobilisation was less apparent for the pGLP-1 at the human receptors (Fig. 6). These observations suggest that monotreme GLP-1 peptides may have gained new as yet undefined functions.

### Platypus GLP-1 stimulates insulin release in mouse islet cells

Ultimately, the signal cascade triggered by incretins results in the release of insulin from pancreatic islet cells. We investigated the ability of pGLP-1 to stimulate insulin release from isolated mouse islets. Results showed that 100 nM pGLP-1 can stimulate insulin release *in vitro* similar to hGLP-1 (Fig. 6f). It appears at least at mouse islet GLP-1R that pGLP-1 would act with classical incretin function to promote insulin release, although whether this is the primary function in the platypus remains to be proven.

## Discussion

Incretin hormones play a key role in regulation of mammalian metabolism through regulation of insulin secretion as well as by decreasing gastric emptying, food intake, body weight and increasing satiety. The importance of insulin regulation and metabolic control has led to recruitment of genes in this pathway in venom function in various species. Of medical importance, the discovery of the DPP-4 protease resistant *exendin-4* from venom of *Heloderma suspectum* paved the way for the successful development of GLP-1 analogues as important treatments for insulin resistant type 2 diabetics.

Monotremes are a fascinating species to investigate this as they represent the third and most basal lineage of extant mammals. Importantly they have undergone remarkable changes of their digestive system and feature production of potent venom for intraspecific conflict during the breeding season. This prompted us to investigate the incretin hormone GLP-1, its receptor (GLP-1R) and the GLP-1 regulatory enzyme DPP-4 in monotremes. Overall the key genes in this pathway are conserved in monotremes showing that the insulin release pathway has been maintained despite the extensive changes in the digestive system. However, we discovered remarkable changes in GLP-1 sequence and function, which we propose are the result of an unusual evolutionary trajectory of this important gene. When we compared platypus and echidna *GCG* we discovered more changes than would be expected given that monotremes diverged only 17–48 Million years ago. This accelerated rate in evolutionary change maybe driven by new roles for this gene.

Indeed we discovered that the same monotreme *Gcg* gene encoding GLP-1 is expressed in monotreme gut and venom. In contrast, Ex-4 is only found in the venom of the lizard *Heloderma suspectum*, with the endogenous GLP-1 being DDP-4 sensitive and 84% identical at the amino acids level to human GLP-1^14^,^15^. Further changes involving receptor affinity and activation occurred specifically in monotremes. The GLP-1 peptide found in all other mammalian species binds the human GLP-1R with an IC50 of 7.9 × 10^−10^ M (Table 2), whereas the monotreme GLP-1 peptides bind the human and platypus GLP-1R with 50 fold lower affinity. As human GLP-1 is able to bind with high affinity and potently activate the platypus GLP-1R (Fig. 6 and (Supplementary Fig. S6), we conclude that the platypus receptor functions in a similar manner to hGLP-1R. The significance of the low pGLP-1R binding affinity for monotreme GLP-1 peptides to biological activity is as yet unclear.

The phenomenon of ligand-directed signalling bias for GLP-1R has recently been described using the endpoints of cAMP production, pERK1/2 and intracellular Ca^2+^. For example, hGLP-1 and OXM exhibit bias for cAMP over pERK1/2, whereas Ex-4 and GLP-1 [7–37] are not biased. In platypus and echidna the low pGLP-1R binding promote a bias towards pERK1/2 signalling compared to human GLP-1. This indicates that the differences in monotreme sequences promote a change in the way the peptides interact with the GLP-1R and engendering the receptor with a unique conformation to drive distinct receptor function. It also suggests that monotreme GLP-1 peptides might promote unique, as yet unknown functions through the montreme GLP-1Rs. It will be interesting to understand the functional consequences of these changes, which are relevant for possible application in the development of GLP-1R agonists for medical application in diabetes treatment and for better understanding of the function of these changes in monotreme GLP-1. Lineage specific changes in the sequence and function have been described in hystricomorph rodents for the key metabolic gene insulin^31^. However, monotremes present the first example were such a gene, *GCG* in this case, appears to maintain its role in gut as well as adopting a new role in venom. The fact that the venom is used towards members of the same species during the mating season may have favored the use of the endogenous gene as the most effective venom component. This may have set the scene for an adaptive conflict where on one side selection would favour tolerance to a spike in GLP-1 in blood as a result of envenomation and on the other increased potency as a venom component. At the same time insulin releasing function of GLP-1 has to be maintained. Insulinotropic effects associated with GLP-1-like peptides have been reported in a range of venomous species including arthropods, reptiles^32^,^33^,^34^,^35^,^36^, and recently in cone snail venom, which induces severe hypoglycemic shock in its fish prey^16^. In contrast to the evolution of *Gcg*-like genes and insulin mimetics in other species, monotremes are the first examples of species that have recruited the *endogenous* GLP-1 system into venom. The fact that monotreme GLP-1 is also expressed in monotreme venom raises the possibility that DPP-4 resistance is selected for when GLP-1 like molecules are recruited into venom function. However, monotremes use venom during intraspecific conflict rather than for envenomation of prey, as is the case for most other venomous species. While the DPP-4 resistance is likely a change to enhance the insulinotropic effect, the changes in the degradation system may have evolved as a countermeasure to the venom’s effect mediated by GLP-1 on blood glucose. Further more, it may be that the decreased affinity for the receptor has also evolved as a protective mechanism in response to the use of GLP-1 in venom.

Clearly the insulin releasing property of GLP-1 is common in tetrapods but its function in venom is novel and monotreme specific. Such acquisition of new function, termed moonlighting, is observed in many genes. However, if a newly acquired function results in selective conflict it has been postulated that gene duplication is favoured^37^. Alternatively functional diversification can be the result of duplication events, which maybe the case for the many venom genes, including *exendin*s that are likely to be the result of gene duplication involving *GCG* and *GIP*^9^,^14^. In monotremes the changes observed indicate that there is selective conflict between GLP-1’s function in venom and its traditional function. It is therefore surprising that *GCG* gene duplication has not been selected for. However, gene duplication would mean an increase in gene dosage, which would likely be deleterious as insulin release and glucose tolerance is sensitive to GLP-1 dosage^38^,^39^.

In summary, we propose that in monotremes an evolutionary arms race between the function of GLP-1 in gut and in venom can explain the changes observed. Evolution of DPP-4 independent GLP-1 degradation and decreased receptor activation may have evolved in response to GLP-1 in venom. The independent evolution of these components affecting glucose homeostasis and insulin release also highlights the importance of metabolic control as a target for venomous species. This maybe the first example of a gene where selective conflict has not favoured the evolution of gene duplication.

## Methods

### Materials

For tissue culture Dulbecco’s modified Eagle’s medium (DMEM), RPMI-1640 medium, hygromycin-B, and Fluo-4 acetoxymethyl ester were used (Invitrogen Carlsbad, CA). AlphaScreen reagents, ^125^I-Ex(9–39), ^125^I-hGLP-1 and 384-well ProxiPlates were purchased from PerkinElmer Life and Analytical Sciences (Waltham, MA). hGLP-1, pGLP-1, eGLP-1 and Ex-4 were purchased from GL Biochem (Shanghai) Ltd. (Shanghai, China). All other reagents were purchased from Sigma-Aldrich (St. Louis, MO) or BDH Merck (Melbourne, VIC, Australia) and were of an analytical grade. The parental INS-1 (832/13) cell line was kindly provided by Chris Newgard^40^.

### RNA extraction

Platypus and echidna tissues were obtained from adult animals in accordance with ethics guidelines (approved by Adelaide University Animal Ethics Committee permit AEC S-49–200 to F.G). Total RNA was extracted from snap frozen platypus tissues (frontal cortex, pancreas, liver, lung, small intestine, stomach, heart, venom gland, testis, muscle, lymph and kidney) and echidna tissue (small intestine, pancreas, liver, venom gland, heart and brain) using TRIzol (Invitrogen, USA) according to the manufacture’s instructions. RNA was resuspended in nuclease free water and stored at −80 °C.

### Phylogeny

An evolutionary comparison was made of monotreme *GCG* genes with orthologues in other vertebrate species (encoded by *GCG* genes listed in Supplementary Table S2). The phylogenetic tree was constructed based on the preproglucagon multiple amino acid sequence alignment using ClustalW2^41^. Neighbor Joining algorithm with bootstrap analysis using 1000 replicates was conducted in MEGA4 software using standard settings^42^. The evolutionary distances were computed using the Poisson correction method and are in the units of the number of amino acid substitutions per site^43^. The analysis involved 9 amino acid sequences. All positions containing gaps and missing data were eliminated.

### cDNA synthesis

cDNA was synthesized from 3 μg RNA with Superscript III Reverse Transcriptase (Invitrogen) following the manufacture’s instructions. Briefly, RNAs were treated with DNase I (Roche) to remove genomic DNA, incubated with 50 ng of random hexamers and 0.5 μl of 10 mM dNTPs for 5 min at 65 °C. After incubation, 2 μl of 5 × First-strand RT buffer, 0.5 μl of 0.1 M dithiothreitol (DTT), 0.5 μl of RNaseOUT™ (40 U/μl), and 0.5 μl SuperScript III Reverse Transcriptase (200 U/μl) were added and incubated at 25 °C for 10 min, and then 50 °C for 50 min, followed by the final termination at 85 °C for 5 min. Finally, 0.2 μl of RNase H (Biolabs, 5 U/μl) were added to each tube and incubated at 37 °C for 20 min. cDNAs were stored at −20 °C.

### RT-PCR

RT-PCR was performed to detect the presence of *Gcg*, *Dpp-4* and *Glp-1r* mRNA in different platypus and echidna tissues. Gene-specific primers (*pGcg*, *pGLP1R* and *pDPP-4* sense and anti-sense primers in Supplementary Table S1) were designed based on the known coding sequences of *pGcg*, *pGlp-1r* or *pDpp-4*. Amplification cycles were: initial denaturation at 94 °C for 3 min, followed by 35 cycles of denaturation at 94 °C for 30 s, annealing at 55 °C for 30 s, and extension at 72 °C for 1 min, followed by a final extension at 72 °C for 7 min. PCR products were run on 1.5% agarose gels and then visualized with ethidium bromide. The identity of PCR products were determined by DNA sequencing.

### DPP-4 enzyme assays

The enzyme assay was performed in flat-bottom 96- well microplates, where each reaction consisted of 100 μL of either 12.5% human, platypus or echidna serum, 0.5 mM of H-Ala-Pro-p-nitroanilide (Bachem) as a substrate in the presence/absence of 100 μM of DPP-4 inhibitor P32/98 (Santa Cruz Biotech) in 100 mM sodium phosphate buffer (PH 7.4) supplemented with 150 mM NaCl and 0.2% Tween-20 with a total volume of 200 μM. Reactions were performed at 37 °C. The absorbance of the released p- nitroanilide was measured every 2 min over 4 h at 405 nm using a Thermo Scientific Multiskan Ascent microplate reader. Enzyme activity was shown as fold changes in absorbance per minute normalized to substrates only group.

### DPP-4 cleavage of GLP-1

hGLP-1, pGLP-1, eGLP-1 and Ex-4 (30 μM) were incubated with DPP-4 (3 U/L) in sodium phosphate buffer (0.1 M, pH 7.4) for 0, 30, 60, 90 and 120 min in 37 °C incubator, respectively, when trifluoroacetic acid (TFA, 0.1%) was added to terminate the reactions. Samples were analysed by RP-HPLC with solvent A (0.1% TFA in water) and B (80% acetonitrile in 0.1% aqueous TFA) at a flow rate of 0.5 ml/min. The peptides were eluted with a linear gradient of solvent B from 35% to 50% for 30 min. Elution of the peptides was detected by measuring UV absorption at 215.8 nm.

### Stability of GLP-1 in human, platypus and echidna serum

Human (or platypus or echidna) serum was purified by DEAE Affi-Gel Blue Cartridges (Bio-Rad). hGLP-1, pGLP-1, eGLP-1 and Ex-4 (7 μM) were incubated with purified human serum in 37 °C incubator in the presence/absence of 100 μM of DPP-4 inhibitor P32/98 (Santa Cruz Biotech), and aliquots of the reaction solutions were extracted after 0, 3, 6, 9 and 11 h incubation respectively, when TFA (0.1%) was added to terminate the reactions. Aliquots were analysed by RP-HPLC. Human serum was from the author Briony Forbes and provided with informed consent. We confirm that all methods for these studies were carried out in accordance with relevant guidelines and regulations, and all experimental protocols satisfy the Adelaide University Human Research Ethics Committee criteria as being “negligible risk research”.

### MALDI mass spectrometry

1 μl of fragments collected from RP-HPLC was sported onto an 800 μm Anchor Chip target plate (Bruker Daltonics, Bremen, Germany) separately and air dried. 1 μl of matrix [alpha-Cyano-4-hydroxycinnamic acid, 0.5 mg/mL in water/acetonitrile/TFA 10/90/0.1] was spotted subsequently and air dried. Mass spectra were acquired on an ultraflex III MALDI-TOF/TOF mass spectrometer (Bruker Daltonics) operating in reflective positive mode. Instrument settings were set in flexControl software (Version 3.4, Bruker Daltonik GmbH). Sample *m/z* range was set to 300–4000 Da. 1000 shots were collected for the external calibration and sample measurement. External calibration was performed using a 1:20 dilution of peptide calibration standard (Bruker Daltonics). Laser intensity and detector gain were manually adjusted for optimal resolution. The MS spectra obtained were analysed using the FlexAnalysis software (Version 3.3, Bruker Daltonics) employing smoothing, background subtraction and peak detection algorithms.

### Circular dichroism spectroscopy (CD)

Far-UV CD spectra were measured on a Jasco J-815 spectropolarimeter (Jasco Inc., Easton, MD) at 20 °C in a 1 mm quartz cuvette. The scanning range was 185–300 nm at a speed of 20 nm/min, the bandwidth was 1 nm, and the spectra were accumulated 5 times. The concentration of hGLP-1, pGLP-1, eGLP-1 and Ex-4 was 75 μM in 10 mM sodium phosphate buffer (pH 7.0). The secondary structure of each peptide was estimated using the CONTIN algorithm^44^,^45^.

### Transfections and cell culture

Human and pGLP-1R cDNAs were isogenically integrated into FlpIn-Chinese hamster ovary (FlpInCHO) cells (Invitrogen) and selection of receptor-expressing cells accomplished by treatment with 600 μg/ml hygromycin B as described previously^46^. Transfected and parental FlpInCHO cells were maintained in DMEM supplemented with 10% heat-inactivated FBS and incubated in a humidified environment at 37 °C in 5% CO_2_. INS-1(832/13) cells were cultured in RPMI-1640 medium supplemented with 10 mM HEPES, 10% heat-inactivated FBS, 2 mM L-glutamine, 1 mM sodium pyruvate, 0.05 mM β-mercaptoethanol, 100 iU/ml penicillin, and 100 g/ml streptomycin at 37 °C in a humidified 5% CO_2_ atmosphere.

### Radioligand binding assay

FlpInCHO-hGLP-1R and FlpInCHO-pGLP-1R cells were seeded at a density of 3 × 10^4^ cells/well and INS-1(832/13) cells at 10^5^ cells/well into 96-well culture plates and incubated overnight at 37 °C in 5% CO_2_. Growth media was replaced with binding buffer [phenol-free DMEM containing 25 mM HEPES and 0.1% (w/v) BSA] containing 0.7 nM ^125^I-Ex(9–39) or 0.15 nM ^125^I-hGLP-1 and increasing concentrations of unlabelled ligand. Cells were then incubated overnight at 4 °C, followed by three washes with ice-cold 1 × PBS to remove unbound radioligand. Cells were then lysed in 0.1 M NaOH, and radioactivity determined by γ-counting as described previously^47^.

### cAMP accumulation assay

FlpInCHO-hGLP-1R and FlpInCHO-pGLP-1R cells were diluted to the density of 6 × 10^5^ cells/ml and INS-1(832/13) cells to 2 × 10^6^ cells/ml in stimulation buffer [phenol-free DMEM containing 5 mM HEPES, 0.5 mM 3-isobutyl-1-methylxanthine (IBMX) and 0.1% (w/v) BSA], and cAMP detection was carried out as described previously^48^. All values were converted to concentration of cAMP using a cAMP standard curve performed in parallel, and data were subsequently normalized to the response of 100 nM forskolin.

### ERK1/2 phosphorylation assay

FlpInCHO-hGLP-1R and FlpInCHO-pGLP-1R cells were seeded at a density of 3 × 10^4^ cells/well into 96-well culture plates and incubated overnight at 37 °C in 5% CO_2_. Receptor-mediated ERK1/2 phosphorylation was determined as previously described^47^.

### Intracellular Ca^2+^ mobilization assay

FlpInCHO-hGLP-1R and FlpInCHO-pGLP-1R cells were seeded at a density of 3 × 10^4^ cells/well into 96-well culture plates and incubated overnight at 37 °C in 5% CO_2_ and receptor-mediated intracellular Ca^2+^ mobilization determined as described previously^49^.

### Measurement of insulin

Insulin concentrations were determined by a commercially available radioimmunoassay specific for rodent insulin (Linco Research Immunoassay, St. Charles, MO) as previously described^50^.

### Data analysis

All data were analysed in Prism 6.0c (GraphPad Software Inc., San Diego, CA). Concentration response signalling data were analysed using a three-parameter logistic equation as described previously^46^,^47^. Signalling bias was analysed as described^51^. Briefly, quantification of signal bias was performed using pharmacologically derived parameters of agonist affinity (Ka) and efficacy (tau) for each ligand in each of the three signalling pathways (cAMP accumulation, ERK1/2 phosphorylation, Intracellular Ca^2+^ mobilisation). The transduction ratio (tau/Ka) was extracted from standard concentration-response data that was analysed with the operational model of agonism (Kenakin & Christopoulos 2012). This value was used to calculate ΔΔ(tau/Ka) values through normalization of the transduction coefficient (tau/Ka) for each ligand in each signalling pathway to the reference ligand (hGLP1 in black) and the reference signalling pathway (cAMP). Data are presented on a log scale.

Statistical analysis was by One-way ANOVA (nonparametric) with Dunnett’s post test unless otherwise stated in the figure legends.

## Additional Information

**How to cite this article**: Tsend-Ayush, E. *et al*. Monotreme *glucagon-like peptide-1* in venom and gut: one gene – two very different functions. *Sci. Rep.* **6**, 37744; doi: 10.1038/srep37744 (2016).

**Publisher's note:** Springer Nature remains neutral with regard to jurisdictional claims in published maps and institutional affiliations.

## Supplementary Material

Supplementary Dataset

## Figures and Tables

**Table 1:** Percentage of intact peptides remaining after 11 h incubation in each serum.

Peptides	Intact peptide remaining (%)		
	Human Serum	Platypus Serum	Echidna Serum
hGLP-1	9.4 ± 2.3	11.4 ± 2.8	10.8 ± 2.7
pGLP-1	96.4 ± 2.0	55.1 ± 3.5*	49.4 ± 2.9*
eGLP-1	99.3 ± 1.8	48.3 ± 3.5*	42.4 ± 4.3*
Ex-4	98.1 ± 1.0	100.5 ± 1.2	70.5 ± 3.1*

Data were derived by determining the area under the curve following rHPLC analysis.

*Statistically significant, *P* < 0.05 compared with human serum group. All values are means ± S.E.M. of three experiments conducted in triplicate.

**Table 2:** Characterisation of the binding to human and platypus GLP-1R.

Peptides	pIC_50_	
	hGLP-1R CHO	pGLP-1R CHO
hGLP-1	9.1 ± 0.1	8.9 ± 0.1
pGLP-1	7.9 ± 0.1^*^	7.4 ± 0.1^*,&^
eGLP-1	7.6 ± 0.1^*,#^	7.4 ± 0.1^*^
Ex-4	9.2 ± 0.1	9.2 ± 0.1
OXM	7.4 ± 0.1^*^	7.5 ± 0.1^*^

Data were analysed with a three-parameter logistic equation. pIC_50_ values represents the negative logarithm of the concentration of agonist that inhibits binding of half the total concentration of radiolabelled agonist ^125^I-hGLP-1 (7–36). All values are means ± S.E.M. of two or three experiments conducted in triplicate. ^*^Statistically significant, p < 0.05 compared with hGLP-1, ^#^Statistically significant, *P* < 0.05 when comparing eGLP-1 with pGLP-1, ^&^Statistically significant, *P* < 0.05 when compared with binding to hGLP-1R (paired t test).

**Table 3:** Characterisation of activation of human and platypus GLP-1R.

Peptides	pEC_50_						
	hGLP-1R CHO			pGLP-1R CHO			INS-1 (832/13)
	cAMP accumulation	Ca^2+^ mobilization	pERK1/2	cAMP accumulation	Ca^2+^ mobilization	pERK1/2	cAMP accumulation
hGLP-1	10.5 ± 0.1	7.8 ± 0.1	8.8 ± 0.1	10.7 ± 0.1	8.3 ± 0.1^&^	8.9 ± 0.1	9.6 ± 0.3
pGLP-1	8.7 ± 0.1^*^	6.5 ± 0.1^*^	7.9 ± 0.1^*^	8.8 ± 0.1^*^	7.0 ± 0.1^*,&^	8.4 ± 0.1^&^	7.6 ± 0.3^*^
eGLP-1	6.9 ± 0.1^*,#^	5.0 ± 0.2^*,#^	6.1 ± 0.1^*,#^	6.8 ± 0.1^*,#^	5.0 ± 0.2^*,#^	6.3 ± 0.1^*,#^	6.1 ± 0.3^*,#^
Ex-4	11.1 ± 0.1^*^	8.4 ± 0.1^*^	9.0 ± 0.1	11.1 ± 0.1	8.1 ± 0.1	9.2 ± 0.1	10.3 ± 0.2
OXM	8.0 ± 0.1^*^	6.4 ± 0.1^*^	7.8 ± 0.1^*^	7.8 ± 0.1^*^	6.4 ± 0.1^*^	8.1 ± 0.1^*^	8.6 ± 0.3

Data were analysed with a three-parameter logistic equation as defined in refs 46, 47 and 49, where pEC50 values represent the negative logarithm of the concentration of agonist that produces half the maximal response. All values are means ± S.E.M. of at least three experiments conducted in triplicate. ^*^Statistically significant, p < 0.05 compared with hGLP-1; ^#^Statistically significant, p < 0.05 when comparing eGLP-1 with pGLP-1. ^&^Statistically significant, p < 0.05 when compared with data on the hGLP-1R (paired t test).
