# Supplementary material for: Monotreme glucagon-like peptide-1 in venom and gut: one gene – two very different functions
Source: Sci Rep. 2016 Nov 29;6:37744. doi: 10.1038/srep37744 (PMC5127184; doi:10.1038/srep37744)
Supplement: Supplementary Dataset [file srep37744-s1.doc]

Supplementary Information

**Monotreme *glucagon-like peptide-1* in venom and gut: one gene – two very different functions**

(short title: Novel GLP-1 functions in monotremes)

**Enkhjargal Tsend-Ayush^*a^, Chuan He^*a^**, **Mark Myers^b^, Sof Andrikopoulos^c^, Nicole Wong^c^, Patrick M Sexton^d^, Denise Wootten^d^, Briony E Forbes^**a,e^ and Frank Grutzner^**a^**

*Author Affiliation:*

^a^ Robinson Research Institute, School of Biological Sciences, The University of Adelaide, South Australia, 5000, Australia.

^b^ School of Health Sciences, Federation University Australia, Mount Helen, Victoria, 3353, Australia.

^c^ Department of Medicine, Austin Health, The University of Melbourne, Heidelberg, Victoria 3084, Australia.

^d^ Monash Institute of Pharmaceutical Sciences and Department of Pharmacology, Monash University, Parkville, Victoria, 3052, Australia.

^e^ School of Medicine, Flinders University, Bedford Park, South Australia, 5042, Australia

***equal first author contribution**

****equal last and corresponding authors**

**Contents**

**Supplementary Tables S1 and S2**

**Supplementary Figures S1-S6**

**Supplementary References**

| **Supplementary Table S1**  **Primers used in this study.** | | | |
| --- | --- | --- | --- |
|  |  | Primer sequences | Fragment size (bp) |
| *pGcg* | sense | 5'-TGG TCC GAT TAC CAA ACG TCA-3' | 350 |
|  | anti-sense | 5'-TCA CCT CTC CTC AAC TTC GG-3' |  |
| *eGcg* | sense | 5'-TGG CTG GAT TGT TCG TGA TG-3' | 360 |
|  | anti-sense | 5'-GCT GCC GCT ACC TCT CTT GA-3' |  |
| *pGlp-1r* | sense | 5'-GAC GAC TAC GCC TGT TGG-3' | 404 |
|  | anti-sense | 5'-GAG CAC GGT GTC CTT GAT GA-3' |  |
| *Dpp-4* | sense | 5’-GCA AAC CAG GAA ATA GGC AGT G-3’ | 402 |
|  | anti-sense | 5‘-TCT GAA CTC GCA AGG GAG GTA G-3' |  |
| *β-actin* | sense | 5'-GCC CAT CTA CGA AGG TTA CGC-3' | 348 |
|  | anti- sense | 5'-AAG GTC GTT TCG TGG ATA CCA C-3' |  |

**Supplementary Table S2**

**Accession numbers of genes analysed/generated in this study.**

| **Species** | **GCG** | **GLP-1R** | **DPP-4** |
| --- | --- | --- | --- |
| Xenopus | ENSXETP00000028848 |  |  |
| Chicken | ENSGALP00000018068 |  |  |
| Echidna | KX589237^a^ |  |  |
| Platypus | KX589236^a^ | ENSOANP00000020797 | ENSOANP00000009893 |
| Opossum | ENSMODP00000006882 |  | ENSMODP00000038697 |
| Mouse | ENSMUST00000102733 |  |  |
| Guinea pig | ENSCPOP00000014570 |  |  |
| Horse | ENSECAP00000004119 |  |  |
| Human | ENSP00000387662 | ENSP00000362353 | ENSP00000353731 |

^a^ sequences derived in this study

**Supplementary Figure S1**

**Supplementary Figure S1. Amino acid sequence comparisons of a range of DPP-4 substrates (including peptide hormones, neuropeptides and chemokines) in human and platypus.** Identical residues are highlighted in grey boxes. Platypus glucagon, GLP-1 and GLP-2 peptide sequences are from our transcript sequence submitted to Genbank KX589236. The red dashed line indicates DPP-4 cleavage sites. The percentage of similarity between platypus and human homologs is shown. For high cleavage efficiency, hydrophobic and basic residues (e.g., Tyr and His) at N-terminus and Ala or Pro in the penultimate position are preferred^1^. Some peptides with Gly, Ser (e.g., glucagon^2^), Val or Leu in the penultimate P1’ position are cleaved by DPP-4 at very low rates^1^. The different penultimate residue (Ala rather than Ser as in humans) of pGLP-1 and pGIP renders these two peptides DPP-4 resistant.

**Supplementary Figure S2**

**Supplementary Figure S2. Sequence alignment of platypus and echidna glucagon and GLP-2 peptides.** Peptide sequences derived from monotreme cDNA transcripts sequenced in this study are aligned against human sequences. Residues with shared identity are highlighted with grey boxes. The platypus and echidna glucagon sequences differ from human glucagon by 24% and 17% respectively, whereas the platypus and echidna GLP-2 sequences differ from human GLP-2 by 51% and 45% respectively.

**Supplementary Figure S3**


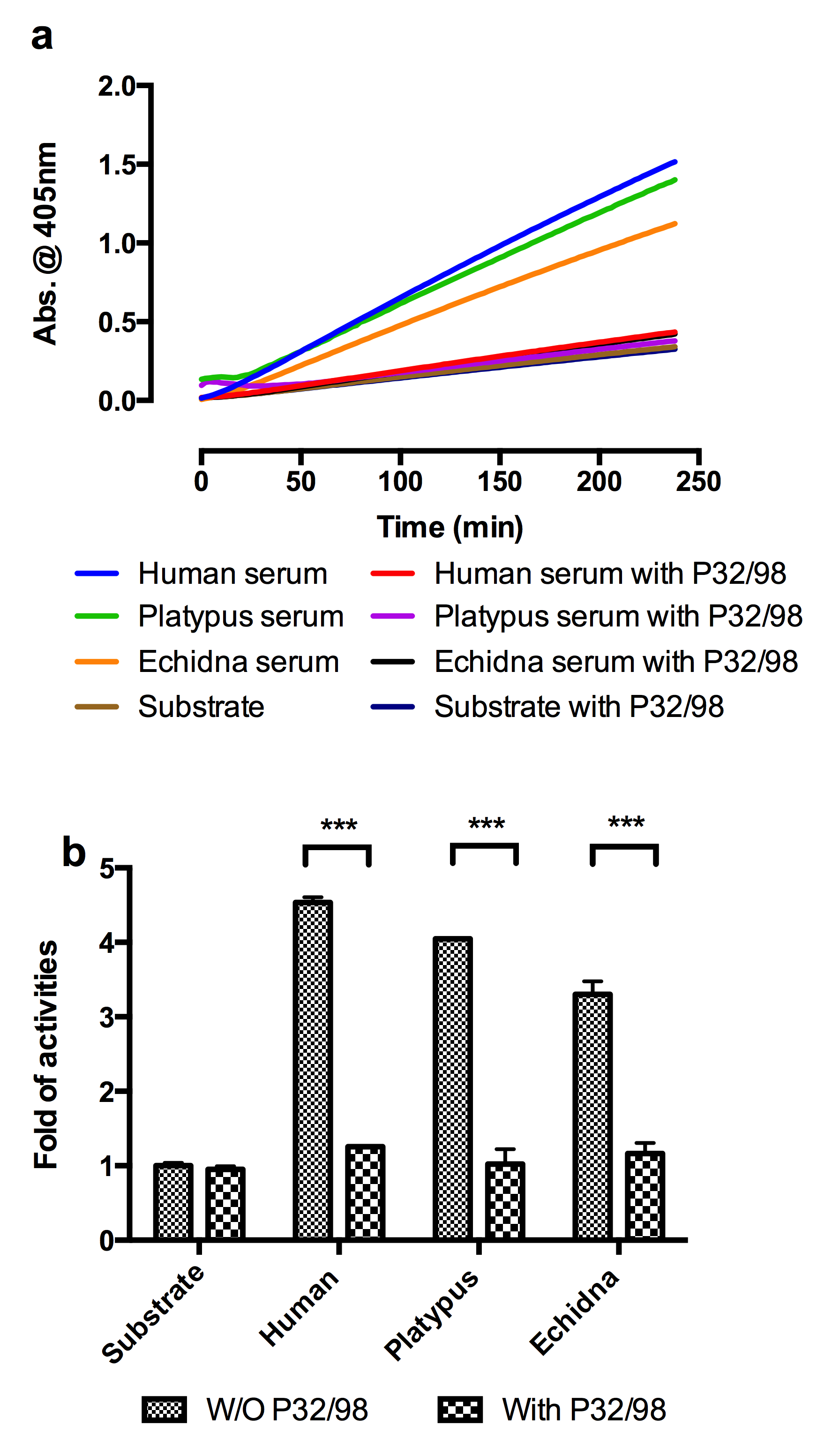


**Supplementary Figure S3. Inhibition of DPP-4 activity in human, platypus and echidna serum by the DPP-4 inhibitor P32/98 monitored during the enzyme assay.** (a) 12.5% human, platypus or echidna serum was incubated with H-Ala-Pro-p-nitroanilide as a substrate in the presence/absence of 100µM DPP-4 inhibitor P32/98 at 37°C. The absorbance of the released p-nitroanilide was measured every two minutes over four hours at 405 nm and is directly related to DPP-4 cleavage activity. (b) DPP-4 activity in each serum with or without inhibitors is shown as fold change in absorbance per minute normalized to the substrate only group. *** Statistically significant, *P* < 0.001 versus groups without inhibitor.

**Supplementary Figure S4
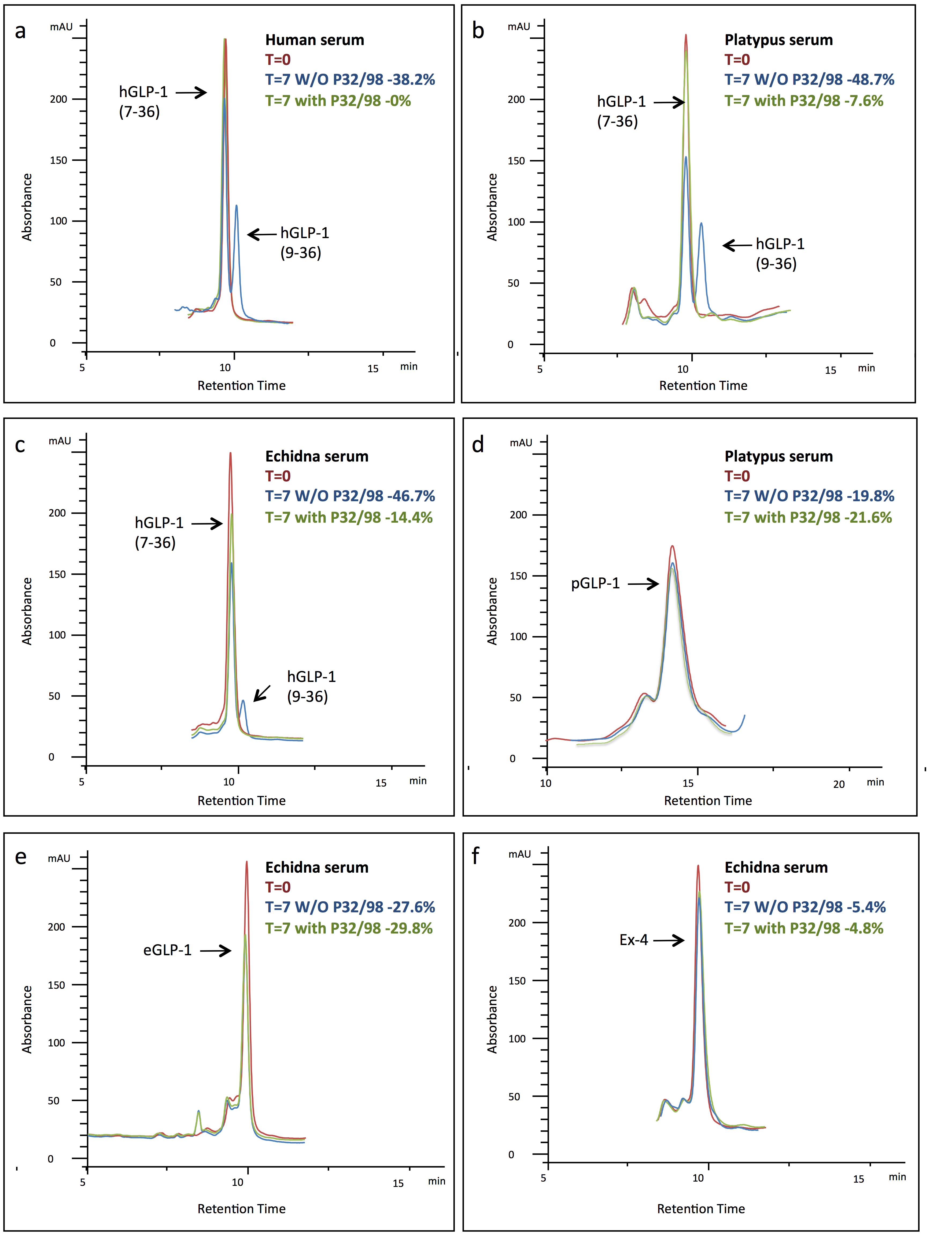
**

**Supplementary Figure S4. Degradation of GLP-1 and Ex-4 by human, platypus or echidna serum monitored by RP-HPLC in the absence and present of DPP-4 inhibitor (P32/98).** Degradation of hGLP-1 by human (a), platypus (b) or echidna serum (c) monitored by RP-HPLC in the absence (blue line) and presence (green line) of a DPP-4 inhibitor (P32/98). In human serum, hGLP-1 remained intact (0% degraded) in the presence of DPP-4 inhibitor, whereas in monotreme sera the inhibitor failed to completely protect the hGLP-1 DPP-4 degradation, indicating the possibility of existence of both DPP-4 and additional enzymes that can degrade hGLP-1 in monotreme sera. This was particularly evident in echidna serum (c), where 14% of the hGLP-1 was degraded despite the presence of inhibitor. Degradation by platypus serum of pGLP-1 (d), eGLP-1 (e) and Ex-4 (f) in echidna serum was monitored by RP-HPLC in the absence (blue line) and presence (green line) of DPP-4 inhibitor (P32/98). The presence of DPP-4 inhibitor in serum did not alter the cleavage pattern, indicating enzymes other than DPP-4 may be responsible for pGLP-1, eGLP-1 and Ex-4 degradation in monotreme serum.

**Supplementary Figure S5**


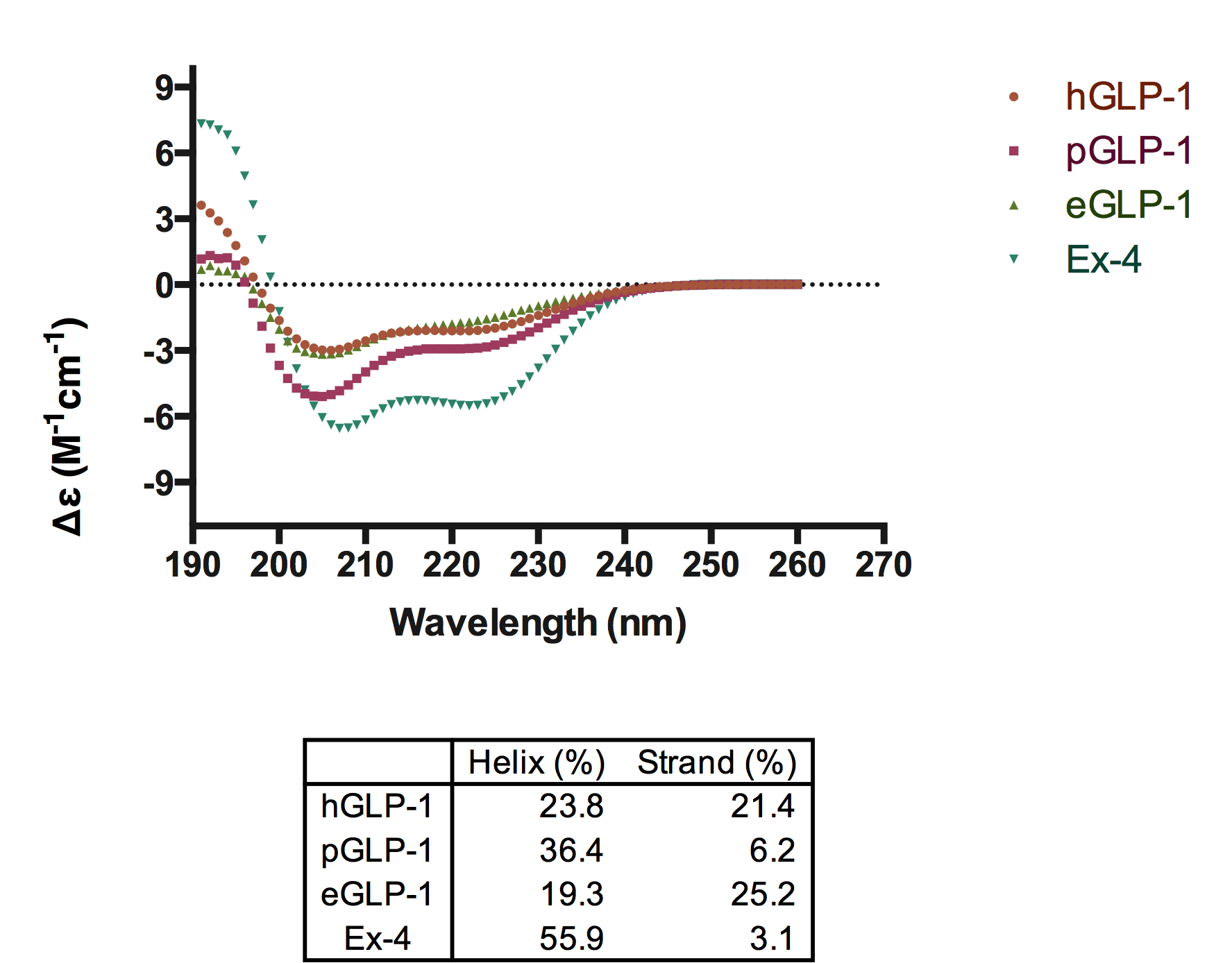


**Supplementary Figure S5. CD spectroscopy of hGLP-1 and analogs.** hGLP-1 (orange circles), pGLP-1 (pink squares), eGLP-1 (green triangle) and Ex-4 (turquoise inverted triangles) (75µM). The secondary structure content of each peptide was estimated using the CONTIN algorithm^3,4^.

**Supplementary Figure S6**

**
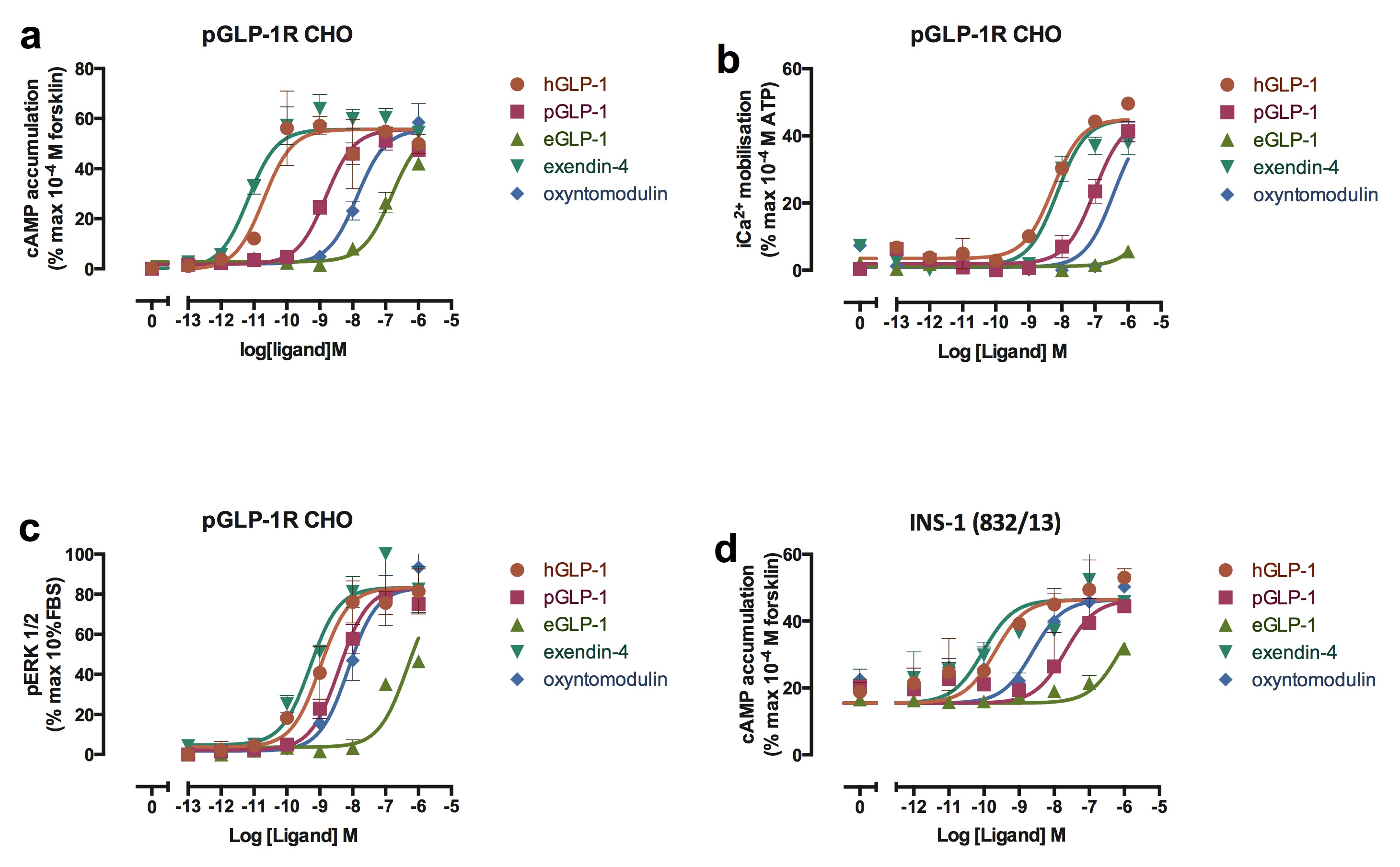
**

**Supplementary Figure S6. Characterisation of the signalling outcomes stimulated by hGLP-1, pGLP-1, eGLP-1, Ex-4 and OXM** in FlpInCHO cells stably expressing pGLP-1R via cAMP accumulation (a), Ca^2+^ mobilization (b) and ERK1/2 phosphorylation (c) and in INS-1 (832/13) cells via cAMP accumulation (d). Data were normalized to the maximal response induced by 100µM forsklin (cAMP), 100µM ATP (Ca^2+^) or 10% FBS (ERK1/2). Data were analysed with a three-parameter logistic equation as defined in ^5,6^. All values are means ± S.E.M. of at least three experiments conducted in triplicate.

**Supplementary Figure S7**

**hGLP-1R**

**pGLP-1R**

**hGLP-1R**

**pGLP-1R**

**A**

**B**

**C**

**D**

**E**

**F**

**Supplementary Figure S7. Stimulation bias of monotreme GLP-1 peptides at the hGLP-1R and pGLP-1R compared to the reference ligand hGLP-1:** Data were analyzed using an operational model of agonism as previously ^7^ to estimate log tc/KA ratios. Changes in log tc/KA ratios were calculated to provide a measure of the degree of stimulus bias (ΔlogRn (Bias factor) exhibited between different signaling pathways relative to that of the reference agonist hGLP-1. Values are expressed as means ± S.E.M. of at least three independent experiments conducted in triplicate. Data were analyzed with one-way analysis of variance and Dunnett’s post test. *Statistically significant at *P* < 0.05 versus negative control group without peptide.

**Supplementary References**

1 Mentlein, R., Gallwitz, B. & Schmidt, W. E. Dipeptidyl-peptidase IV hydrolyses gastric inhibitory polypeptide, glucagon-like peptide-1(7-36)amide, peptide histidine methionine and is responsible for their degradation in human serum. *Eur J Biochem* **214**, 829-835 (1993).

2 Hinke, S. A. *et al.* Dipeptidyl peptidase IV (DPIV/CD26) degradation of glucagon. Characterization of glucagon degradation products and DPIV-resistant analogs. *J. Biol. Chem.* **275**, 3827-3834 (2000).

3 Sreerama, N. & Woody, R. W. Estimation of protein secondary structure from circular dichroism spectra: comparison of CONTIN, SELCON, and CDSSTR methods with an expanded reference set. *Anal. Biochem.* **287**, 252-260, doi:10.1006/abio.2000.4880 (2000).

4 Provencher, S. W. & Glockner, J. Estimation of globular protein secondary structure from circular dichroism. *Biochemistry (Mosc).* **20**, 33-37 (1981).

5 Koole, C. *et al.* Polymorphism and ligand dependent changes in human glucagon-like peptide-1 receptor (GLP-1R) function: allosteric rescue of loss of function mutation. *Molecular pharmacology* **80**, 486-497, doi:10.1124/mol.111.072884 (2011).

6 May, L. T. *et al.* Structure-function studies of allosteric agonism at M2 muscarinic acetylcholine receptors. *Molecular pharmacology* **72**, 463-476, doi:10.1124/mol.107.037630 (2007).

7 Wootten, D. *et al.* Differential activation and modulation of the glucagon-like peptide-1 receptor by small molecule ligands. *Molecular pharmacology* **83**, 822-834, doi:10.1124/mol.112.084525 (2013).
